# Mortality of Philadelphia, for July, August and September, 1854; Collated from the Health Office Record

**Published:** 1854-11

**Authors:** Wilson Jewell


*Mortality of Philadelphia, for July, August and September, 1854; collated from the Health Office record.* By WILSON JEWELL, M.D.

By reference to the accompanying tables, which have been carefully arranged, it will be noticed that the deaths for the quarter ending Sept. 30th, including a period of thirteen weeks, or 91 days, have reached the unusual number of 4,531. This amount will average 49⅔ deaths daily, or, to the population, as ten in every thousand.*

This inordinate increase of deaths, over those for the preceding quarters of the year, almost equal to the two combined, will, upon reflection, be ascribed to its true causes. First, the fact that the record embraces now, as far as they can be secured, the returns from the whole county. Second, the prevalence of Asiatic Cholera during the quarter, from which alone there have been registered 521 deaths.

It will be observed also, that the deaths from Cholera Infantum have contributed a large share towards making up the table. From this disease alone, 621 deaths have been returned, more than twice the number of those for the like quarter of last year.

It would not, however, be proper to charge the deaths from these two diseases upon the additional territory now included within the bounds of the consolidated City, because they are peculiar to densely populated places, and whereas the additions made to our city, embrace principally rural districts. Hence the inference, that the large numerical increase to the register by Cholera Asphyxia and Cholera Infantum at least, has been the result of a sickly season, rather than from the enlargement of the city limits.

By comparison, the increase of deaths over the same quarter for '53 is equal to 34 per cent.

The deaths from authenticated diseases alone number 3,875, while 656 are set down to natural, adventitious and unknown causes.

* This calculation is based upon the population of the consolidated City, which is set down at 450,000.

Infantile mortality for the quarter, occupies, as usual, a very large proportion of the record. Exclusive of Still-born, amounting to 132, there were 1,053 deaths under one year of age. Under five 2,221, nearly one-half the total for the quarter.

The excess of deaths in the male sex is equal to 15 per cent. That of male still-born children 12 per cent.

CLASS 1st. "Endemic and Epidemic" diseases. This division represents 43 per cent. of all the deaths for the quarter; a much higher proportion than has been observed since 1849. Nearly doubling those, in the like class, for the same quarter of the past year, to wit: In 1853 they amounted to 999; this year to 1,962.

Cholera Infantum exhibits a large increase over last year. In 1853, third quarter, there were 308 deaths; this year, 621.

Cholera Asphyxia contributes 521 to swell the record for the quarter, equal to 14 per cent. of all the deaths that have been registered. Last year this disease was scarcely known.

Dysentery has been very prevalent, the deaths numbering 319; so likewise Diarrhœa, from which there were 152 deaths.

Ten hundred and thirty-five of all the deaths in this class were under five years. Showing a frightful proportion of infantile mortality, induced by the prevalence of Cholera Infantum, Dysentery, Diarrhœa and Hooping Cough.

CLASS 2d. From "Sporadic" or "uncertain" diseases there were 539 deaths, an increase of 139 over those of the third quarter, 1853. Of the deaths recorded in this variety, more than two-thirds were under five years of age. Within this early period of life, Marasmus is credited with 221. We have no doubt, however, that many of these cases, if properly assigned, would have been placed to the account of Cholera Infantum.

Debility, under this classification, furnishes 180 deaths, more than half of which were within the fifth year of life. This indefinite term, like some others to be found in our mortuary tables, in many instances a mere symptom or the product of disease, should be applied but seldom.

CLASS 3d. "Nervous System." Here also we mark a considerable increase of deaths over those for a like period in 1853, when there were recorded only 509. Now they are augmented to 781, an increase of 35 per cent.

Of this class, Convulsions in Children make up 227, or nearly 30 per cent. of the whole.

Those diseases of the Brain, marked Congestion, Dropsy and Inflammation, have been more than usually prevalent, sweeping off 319, mostly children—or about 40 per cent. of all the deaths enumerated in this class of diseases.

Class 4th. The deaths from the diseases of the "Organs of Respiration" present an increase of 80 over those of the third quarter of last year.

The principal disease recorded is Consumption of the Lungs, which carried off in three months 340 individuals, or 17 per cent. more than for the same period of 1853, and 25 per cent. over those of 1852.

Of all the deaths for the quarter, Consumption of the Lungs constitutes about 8 per cent.

From the fifth to the tenth distinctive classes, the increase of deaths as compared with those for the like period of '53, is in the aggregate so small, as scarcely to deserve our attention, unless to observe the uniformity by which several of them are characterized.

Class 11th. "Old Age" numbers 62, double those for '53, third quarter. It becomes a question, whether those who die between 60 and 70, or even within the next decade, 70 and 80, are entitled to the reverential appellation—old age.

Forty-one of those recorded, were over 80 years of age, and two of this number were centenarians.

Of "Still-born" children there were 132—less five, when compared with those for 1853, third quarter—while the deaths distinguished by "unknown," in all 124, are double those of the same period of last year.

This class, of "unknown" deaths, is at all times augmented by the report of the Coroner, as we have on a former occasion intimated. An indifference and a want of care on the part of many practitioners in making a diagnosis of their cases, as well as their unwillingness to sustain a correct and scientific nomenclature of diseases, is also another frequent cause. A reform here would rid our mortuary tables of many vague and unmeaning terms, and enable us to preserve a well classified, uniform, and scientific register of deaths.

TABLE NO. I.

*Deaths for the second quarter of 1854 classified.*

| | July | Aug. | Sept | Male. J. | Male. A. | Male. S. | Female. J. | Female. A. | Female. S. | Total. |
|---|---|---|---|---|---|---|---|---|---|---|
| 1 *Endemic & Contagious diseases* | | | | | | | | | | |
| Zymotic or Epidemic | 809 | 808 | 345 | 441 | 411 | 191 | 368 | 397 | 154 | 1962 |
| 2 *Uncertain or general seat* | | | | | | | | | | |
| Sporadic diseases | 200 | 208 | 131 | 109 | 128 | 65 | 91 | 80 | 66 | 539 |
| 3 Nervous system | 340 | 282 | 159 | 204 | 151 | 88 | 136 | 131 | 71 | 781 |
| 4 Organs of Respiration | 188 | 176 | 133 | 91 | 97 | 69 | 97 | 79 | 64 | 497 |
| 5 " Circulation | 21 | 17 | 11 | 12 | 7 | 4 | 9 | 10 | 7 | 49 |
| 6 Digestive organs | 62 | 70 | 51 | 25 | 37 | 28 | 37 | 33 | 23 | 183 |
| 7 Urinary " | 4 | 2 | | 3 | 1 | | 1 | 1 | | 6 |
| 8 Organs of Generation | 12 | 7 | 5 | 1 | | | 11 | 7 | 5 | 24 |
| 9 " Locomotion | 3 | 1 | 6 | 2 | | 2 | 1 | 1 | 4 | 10 |
| 10 Integumentary system | 3 | 1 | | 3 | | | | 1 | | 4 |
| 11 Old age | 19 | 30 | 13 | 6 | 9 | 2 | 13 | 21 | 11 | 62 |
| 12 External causes | 49 | 69 | 40 | 46 | 51 | 33 | 3 | 18 | 7 | 158 |
| Still Born | 41 | 53 | 38 | 25 | 26 | 22 | 16 | 27 | 16 | 132 |
| Unknown | 39 | 47 | 38 | 17 | 21 | 22 | 22 | 26 | 16 | 124 |
| | 1790 | 1771 | 970 | 985 | 939 | 526 | 805 | 832 | 444 | 4531 |

TABLE NO. 2.

1. *Endemic and Contagious Diseases—Zymotic or Epidemic.*

| | Male. | Female. | Under 1 yr. | 1 to 2. | 2 to 5. | 5 to 10. | 10 to 15. | 15 to 20. | 20 to 30. | 30 to 40. | 40 to 50. | 50 to 60. | 60 to 70. | 70 to 80. | 80 to 90. | 90 to 100. | 100 to 110. | Total. |
|---|---|---|---|---|---|---|---|---|---|---|---|---|---|---|---|---|---|---|
| Cholera Asphyx. | 279 | 242 | 6 | 6 | 24 | 19 | 16 | 16 | 108 | 106 | 92 | 66 | 33 | 19 | 5 | 4 | 1 | 521 |
| " infantum | 328 | 293 | 375 | 214 | 30 | 2 | | | | | | | | | | | | 621 |
| " morbus | 44 | 52 | 5 | 2 | 9 | 11 | 7 | | 10 | 14 | 12 | 10 | 12 | 4 | | | | 96 |
| Croup | 18 | 10 | 5 | 10 | 11 | 2 | | | | | | | | | | | | 28 |
| Diarrhœa | 85 | 67 | 56 | 28 | 13 | 6 | 5 | 1 | 7 | 3 | 7 | 5 | 10 | 6 | 4 | 1 | | 152 |
| Dysentery | 170 | 149 | 47 | 61 | 42 | 24 | 8 | 8 | 25 | 24 | 16 | 27 | 20 | 11 | 6 | | | 319 |
| Erysipelas | 7 | 7 | 3 | 2 | | 1 | | 1 | 2 | 2 | 1 | 1 | | | 1 | | | 14 |
| Fever, | 4 | 3 | | | 1 | | | | 3 | 1 | | | | 1 | | 1 | | 7 |
| " Adynamic | 1 | | | | | | | | 1 | | | | | | | | | 1 |
| " Bilious | 9 | 5 | | | 2 | 1 | 1 | | 1 | 3 | 4 | 2 | | | | | | 14 |
| " " Remit. | 1 | 1 | | 1 | | | | | | | | | 1 | | | | | 2 |
| " Congestive | 3 | 3 | | | 1 | | | 1 | 2 | 1 | | | | 1 | | | | 6 |
| " Continued | 1 | 1 | | | | | | | | | | 1 | 1 | | | | | 2 |
| " Intermit. | 2 | 2 | 1 | 1 | 1 | | | | | | | 1 | | | | | | 4 |
| " Malignant | | 2 | | | | 1 | | 1 | | | | | | | | | | 2 |
| " Nervous | 1 | 1 | | | 1 | | | | | 1 | | | | | | | | 2 |
| " Remittent | 7 | 6 | 1 | 1 | 2 | 1 | | | 3 | 3 | | 1 | 1 | | | | | 13 |
| " Scarlet | 9 | | | 3 | 2 | 4 | | | | | | | | | | | | 9 |
| " Typhoid | 26 | 23 | 1 | 3 | 2 | 5 | 3 | 7 | 13 | 9 | 1 | 4 | 1 | | | | | 49 |
| " Typhus | 12 | 12 | 1 | | 1 | 3 | | 3 | 11 | 3 | | 1 | | 1 | | | | 24 |
| " Yellow | 9 | 3 | | | | | 1 | | 8 | 1 | 1 | 1 | | | | | | 12 |
| Hooping Cough | 25 | 26 | 31 | 12 | 8 | | | | | | | | | | | | | 51 |
| Measles | 1 | 6 | | 3 | 3 | 1 | | | | | | | | | | | | 7 |
| Small Pox | 1 | 3 | 1 | 1 | 1 | | | | 1 | | | | | | | | | 4 |
| Syphilis | | 2 | | | | | | | | 2 | | | | | | | | 2 |
| | 1043 | 919 | 533 | 348 | 154 | 81 | 41 | 38 | 195 | 173 | 134 | 120 | 79 | 43 | 16 | 6 | 1 | 1962 |

*2. Uncertain or General Seat—Sporadic Diseases.*

| | Male. | Female. | Under 1 yr. | 1 to 2. | 2 to 5. | 5 to 10. | 10 to 15. | 15 to 20. | 20 to 30. | 30 to 40. | 40 to 50. | 50 to 60. | 60 to 70. | 70 to 80. | 80 to 90. | 90 to 100. | 100 to 110. | Total. |
|---|---|---|---|---|---|---|---|---|---|---|---|---|---|---|---|---|---|---|
| Abscess | 3 | | | | | | | | 3 | | | | | | | | | 3 |
| " Lumbar | 2 | | | | | | | | 1 | 1 | | | | | | | | 2 |
| " Psoas | 2 | 1 | | | | | | | 1 | 1 | 1 | | | | | | | 3 |
| " Thigh | | 1 | | | | | | | 1 | | | | | | | | | 1 |
| Aphthæ | | 1 | | 1 | | | | | | | | | | | | | | 1 |
| Cachexia | 4 | | 1 | | 1 | | | | 1 | | | | 1 | | | | | 4 |
| Cancer | 2 | 5 | | | | | | | | 1 | 2 | 1 | 3 | | | | | 7 |
| " Breast | | 1 | | | | | | | | | | | | | | 1 | | 1 |
| Caries | | 1 | | 1 | | | | | | | | | | | | | | 1 |
| Cyanosis | 5 | 3 | 7 | | 1 | | | | | | | | | | | | | 8 |
| Debility | 96 | 84 | 76 | 12 | 5 | | 2 | 1 | 4 | 11 | 15 | 14 | 15 | 13 | 10 | 1 | 1 | 180 |
| Disease of Hip | 1 | | 1 | | | | | | | | | | | | | | | 1 |
| Dropsy | 16 | 13 | 3 | 1 | 1 | 2 | 1 | | 3 | 2 | 4 | 2 | 5 | 4 | 1 | | | 29 |
| Enlargem't parot. glands | | 1 | | | 1 | | | | | | | | | | | | | 1 |
| Fungus Hematodes | 2 | | | | 1 | | | | | | 1 | | | | | | | 2 |
| Gangrene | | 1 | | | | | | | 1 | | | | | | | | | 1 |
| Hemorrhage | 3 | 1 | 2 | 1 | | | | | | | 1 | | | | | | | 4 |
| Inanition | 9 | 11 | 11 | 3 | 3 | | | 1 | 1 | | | | | 1 | | | | 20 |
| Inflammation | 2 | 2 | | 1 | 1 | | 1 | | 1 | | | | | | | | | 4 |
| " Fauces | | 1 | 1 | | | | | | | | | | | | | | | 1 |
| Malformation | 4 | 1 | 5 | | | | | | | | | | | | | | | 5 |
| Marasmus | 130 | 91 | 113 | 73 | 23 | 4 | | | 3 | | | | 5 | | | | | 221 |
| Obesity | 1 | | | | | | | | | | | | | 1 | | | | 1 |
| Schirrhus | | 1 | | | | | | | 1 | | | | | | | | | 1 |
| Scrofula | 7 | 3 | 2 | 1 | | 1 | | 2 | 2 | 1 | | 1 | | | | | | 10 |
| Sore Mouth | 3 | 1 | 4 | | | | | | | | | | | | | | | 4 |
| " Throat | | 1 | | | | | | | 1 | | | | | | | | | 1 |
| Tabes Mesenterica | 9 | 10 | 8 | 7 | 3 | 1 | | | | | | | | | | | | 19 |
| Ulceration of Throat | 1 | 2 | | | 3 | | | | | | | | | | | | | 3 |
| | 302 | 237 | 234 | 101 | 43 | 8 | 4 | 4 | 24 | 17 | 24 | 18 | 29 | 19 | 11 | 2 | 1 | 539 |

### 3. *Nervous Diseases.*

| | Male. | Female. | Under 1 yr. | 1 to 2. | 2 to 5. | 5 to 10. | 10 to 15. | 15 to 20. | 20 to 30. | 30 to 40. | 40 to 50. | 50 to 60. | 60 to 70. | 70 to 80. | 80 to 90. | 90 to 100. | 100 to 110. | Total. |
|---|---|---|---|---|---|---|---|---|---|---|---|---|---|---|---|---|---|---|
| Apoplexy | 24 | 15 | | | | | 1 | | 7 | 6 | 8 | 2 | 6 | 8 | 1 | | | 39 |
| Brain Fever | | 1 | | | | 1 | | | | | | | | | | | | 1 |
| Concussion of Brain | 2 | 1 | | | 1 | | | | | | 1 | 1 | | | | | | 3 |
| Congestion " | 51 | 44 | 29 | 16 | 11 | 2 | | 4 | 8 | 8 | 12 | 1 | 3 | 1 | | | | 95 |
| Convulsions | 115 | 112 | 114 | 75 | 23 | 2 | 3 | | 6 | 1 | 3 | | | | | | | 227 |
| " Puerperal | | 2 | | | | | | | | 2 | | | | | | | | 2 |
| Coma | | 2 | | | | | | | | | | | 1 | | 1 | | | 2 |
| Coup de Soleil | 30 | 5 | | | | | | | 16 | 7 | 10 | 2 | | | | | | 35 |
| Dementia | 1 | | | | | | | | | | | | 1 | | | | | 1 |
| Disease of Brain | 18 | 11 | 10 | 6 | 6 | 1 | | | 1 | 1 | 2 | | | 1 | 1 | | | 29 |
| Dropsy " | 58 | 41 | 50 | 32 | 10 | 3 | | 2 | | 1 | | | 1 | | | | | 99 |
| Eclampsia | 1 | | | 1 | | | | | | | | | | | | | | 1 |
| Effusion on Brain | 27 | 13 | 21 | 6 | 5 | 2 | 1 | | 1 | 1 | | 1 | | 1 | 1 | | | 40 |
| Epilepsy | 4 | 4 | | 1 | | | | | 4 | 2 | | 1 | | | | | | 8 |
| Hysteria | | 1 | | | | | | | | | 1 | | | | | | | 1 |
| Insanity | 1 | 1 | | | | | | | 2 | | | | | | | | | 2 |
| Inflammation of Brain | 72 | 53 | 40 | 31 | 22 | 8 | 4 | 1 | 6 | 6 | 1 | 2 | 1 | 2 | 1 | | | 125 |
| Mania | 3 | 3 | | | | | | | 3 | 1 | | | 1 | | | 1 | | 6 |
| " a Potu | 14 | 5 | | | | | | | 6 | 7 | 5 | 1 | | | | | | 19 |
| " Puerperal | | 1 | | | | | | | | 1 | | | | | | | | 1 |
| Nervous Irritation | | 1 | | | | 1 | | | | | | | | | | | | 1 |
| Neuralgia | | 1 | | | | | | | | | | | 1 | | | | | 1 |
| Palsy | 12 | 19 | | 3 | | | | | 1 | 2 | 2 | 3 | 4 | 11 | 4 | 1 | | 31 |
| " of Brain | 1 | | | | | 1 | | | | | | | | | | | | 1 |
| Softening of Brain | 5 | 1 | 1 | | | | | | | 1 | 2 | | 1 | 1 | | | | 6 |
| Tetanus | 3 | | 1 | | 1 | | | | | 1 | | | | | | | | 3 |
| Trismus | 1 | 1 | 2 | | | | | | | | | | | | | | | 2 |
| | 443 | 338 | 268 | 171 | 79 | 21 | 9 | 7 | 61 | 48 | 47 | 14 | 20 | 25 | 9 | 2 | | 781 |

### 4. *Organs of Respiration.*

| | Male. | Female. | Under 1 yr. | 1 to 2. | 2 to 5. | 5 to 10. | 10 to 15. | 15 to 20. | 20 to 30. | 30 to 40. | 40 to 50. | 50 to 60. | 60 to 70. | 70 to 80. | 80 to 90. | 90 to 100. | 100 to 110. | Total. |
|---|---|---|---|---|---|---|---|---|---|---|---|---|---|---|---|---|---|---|
| Abscess of Lungs | 1 | | | | | | | | | | | 1 | | | | | | 1 |
| Asthma | 6 | 2 | 4 | | | | | | | | 1 | 2 | | 1 | | | | 8 |
| Congestion of Lungs | 7 | 6 | 6 | 1 | 3 | | | | | | 2 | | 1 | | | | | 13 |
| Consumption " | 159 | 181 | 14 | 13 | 5 | 4 | 9 | 26 | 96 | 75 | 49 | 28 | 17 | 4 | | | | 340 |
| Disease of Chest | 1 | | | | 1 | | | | | | | | | | | | | 1 |
| " Lungs | 7 | 7 | 4 | 2 | 3 | | | | | 2 | 2 | | 1 | | | | | 14 |
| Dropsy of Chest | 2 | 6 | | | 2 | | | | | 3 | 1 | | 1 | | 1 | | | 8 |
| Fever Hectic | | 1 | | 1 | | | | | | | | | | | | | | 1 |
| Gangrene of Lungs | 1 | | | | | | | | | | | 1 | | | | | | 1 |
| Hemorrhage of " | 3 | 1 | | | 1 | | | | 1 | | 1 | 1 | | | | | | 4 |
| Inflamm'n of Bronchiæ | 15 | 9 | 10 | 7 | 3 | | | | | 2 | | 1 | | 1 | | | | 24 |
| " Larynx | 4 | 1 | 1 | 3 | 1 | | | | | | | | | | | | | 5 |
| " Lungs | 43 | 24 | 18 | 15 | 8 | 1 | 1 | 1 | 6 | 5 | 3 | 2 | 4 | 2 | | 1 | | 67 |
| " Pleura | 1 | 1 | | 1 | | | | | | 1 | | | | | | | | 2 |
| Perforation of Lungs | | 1 | | | | | | | | 1 | | | | | | | | 1 |
| Tuberculosis | 7 | | | 1 | 1 | | | 1 | 2 | | 2 | | | | | | | 7 |
| | 257 | 240 | 57 | 44 | 28 | 5 | 10 | 28 | 105 | 89 | 61 | 36 | 24 | 8 | 1 | 1 | | 497 |

*5. Organs of Circulation.*

| | Male. | Female. | Under 1 yr. | 1 to 2. | 2 to 5. | 5 to 10. | 10 to 15. | 15 to 20. | 20 to 30. | 30 to 40. | 40 to 50. | 50 to 60. | 60 to 70. | 70 to 80. | 80 to 90. | 90 to 100. | 100 to 110. | Total. |
|---|---|---|---|---|---|---|---|---|---|---|---|---|---|---|---|---|---|---|
| Angina Pectoris | 2 | 1 | | | 1 | | | | 1 | | 1 | | | | | | | 3 |
| Aneurism of Aorta | 2 | | | | | | | | 2 | | | | | | | | | 2 |
| Disease of Heart | 8 | 18 | 2 | | 1 | 2 | | 2 | 4 | 5 | 5 | 1 | 1 | 3 | | | | 26 |
| Dropsy " | 4 | 3 | | 1 | | 1 | | | | | | 2 | 1 | 2 | | | | 7 |
| Enlargem't " | 3 | 1 | | | 1 | | | | 2 | | | | 1 | | | | | 4 |
| Inflammat'n " | 1 | 2 | | | | | | | 2 | 1 | | | | | | | | 3 |
| " Veins | 2 | | | | 1 | | 1 | | | | | | | | | | | 2 |
| Malformation of Heart | | 1 | 1 | | | | | | | | | | | | | | | 1 |
| Rupture " | 1 | | | | | | | | | | | 1 | | | | | | 1 |
| | 23 | 26 | 3 | 1 | 4 | 3 | 1 | 2 | 11 | 6 | 6 | 4 | 3 | 5 | | | | 49 |

*6. Digestive Organs.*

| | Male. | Female. | Under 1 yr. | 1 to 2. | 2 to 5. | 5 to 10. | 10 to 15. | 15 to 20. | 20 to 30. | 30 to 40. | 40 to 50. | 50 to 60. | 60 to 70. | 70 to 80. | 80 to 90. | 90 to 100. | 100 to 110. | Total. |
|---|---|---|---|---|---|---|---|---|---|---|---|---|---|---|---|---|---|---|
| Abscess of Liver | 1 | | | | | | | | | | | 1 | | | | | | 1 |
| Cancer of Stomach | 1 | 2 | | | | | | | | | | 1 | | 2 | | | | 3 |
| " Rectum | | 1 | | | | | | | | | 1 | | | | | | | 1 |
| Cancrum Oris | 3 | 1 | | 2 | 2 | | | | | | | | | | | | | 4 |
| Cirrhosis | | 1 | | | | | | | | | 1 | | | | | | | 1 |
| Congestion of Liver | 4 | 1 | 1 | | | | | 1 | | 2 | | | 1 | | | | | 5 |
| Colic | 2 | 1 | 1 | | | | | | 1 | | | | 1 | | | | | 3 |
| Consumption of Bowels | 3 | | 1 | 1 | | | | | | | | 1 | | | | | | 3 |
| Disease of Liver | 2 | 3 | | | | | | | | | 1 | | 2 | 2 | | | | 5 |
| " Stomach | | 1 | | | | | | | | 1 | | | | | | | | 1 |
| " Bowels | 1 | 2 | 1 | | 1 | | | | | | 1 | | | | | | | 3 |
| Dropsy, Abdominal | 6 | 9 | 1 | | | | | 1 | 1 | | 3 | 5 | 2 | | 2 | | | 15 |
| Dyspepsia | 1 | 1 | | | | | | | | | | 1 | 1 | | | | | 2 |
| Hemorrhage of Bowels | | 1 | | | | | | | | | 1 | | | | | | | 1 |
| " Stomach | 2 | | | | | | | | 1 | | 1 | | | | | | | 2 |
| Hernia, Strangulated | 2 | 1 | | | | | | | | | 1 | | | 1 | 1 | | | 3 |
| Inflammation of Bowels | | 1 | | | | | | 1 | | | | | | | | | | 1 |
| " Liver | 3 | 2 | 1 | | | | | | | 1 | 2 | 1 | | | | | | 5 |
| " Stom. & Bowels | 30 | 33 | 18 | 7 | 7 | 1 | | 2 | 4 | 7 | 4 | 2 | 7 | 4 | | | | 63 |
| " Peritoneum | 7 | 7 | 4 | | | | | | 5 | 3 | 1 | 1 | | | | | | 14 |
| Intussusception | | 1 | | | | | 1 | | | | | | | | | | | 1 |
| Jaundice | 3 | 6 | 1 | | 1 | | | | | 2 | 1 | 1 | 2 | 1 | | | | 9 |
| Mortification of Bowels | | 1 | | | | | | | | 1 | | | | | | | | 1 |
| Obstruction of Bowels | 2 | 3 | | | | | | | | | 1 | 2 | 1 | 1 | | | | 5 |
| Schirrus Stomach | | 1 | | | | | | | | | | | | 1 | | | | 1 |
| " Liver | | 2 | | | | | | | | | | | 1 | 1 | | | | 2 |
| Softening of Stomach | 2 | 1 | | 2 | | | | | | | | 1 | | | | | | 3 |
| Teething | 10 | 8 | 8 | 9 | 1 | | | | | | | | | | | | | 18 |
| Ulceration of Stomach | 1 | | | | | | | | | | | 1 | | | | | | 1 |
| " Intestines | 2 | 2 | | 1 | 1 | | | | | | 1 | 1 | | | | | | 4 |
| Worms | 2 | | | 1 | 1 | | | | | | | | | | | | | 2 |
| | 90 | 93 | 37 | 23 | 14 | 1 | 1 | 5 | 12 | 17 | 20 | 19 | 18 | 13 | 3 | | | 183 |

### 7. *Urinary Organs.*

| | Male. | Female. | Under 1 yr. | 1 to 2. | 2 to 5. | 5 to 10. | 10 to 15. | 15 to 20. | 20 to 30. | 30 to 40. | 40 to 50. | 50 to 60. | 60 to 70. | 70 to 80. | 80 to 90. | 90 to 100. | 100 to 110. | Total. |
|---|---|---|---|---|---|---|---|---|---|---|---|---|---|---|---|---|---|---|
| Albuminuria | 1 | 2 | | | | | | | 2 | | | 1 | | | | | | 3 |
| Disease of Kidneys, | 2 | | | | | | | | | | | | 1 | | | 1 | | 2 |
| Inflammation of Bladder, | 1 | | | | | | | | | | | | | 1 | | | | 1 |
| | 4 | 2 | | | | | | | 2 | | | 1 | 1 | 1 | | 1 | | 6 |

### 8. *Organs of Generation.*

| | Male. | Female. | Under 1 yr. | 1 to 2. | 2 to 5. | 5 to 10. | 10 to 15. | 15 to 20. | 20 to 30. | 30 to 40. | 40 to 50. | 50 to 60. | 60 to 70. | 70 to 80. | 80 to 90. | 90 to 100. | 100 to 110. | Total. |
|---|---|---|---|---|---|---|---|---|---|---|---|---|---|---|---|---|---|---|
| Cancer of Uterus | | 2 | | | | | | | | 1 | 1 | | | | | | | 2 |
| Child-bed, | | 2 | | | | | | | | 2 | | | | | | | | 2 |
| Disease of Uterus | | 1 | | | | | | | | | | | 1 | | | | | 1 |
| " Ovary | | 1 | | | | | | | | 1 | | | | | | | | 1 |
| Hemorrhage of Uterus | | 6 | | | | | | | 3 | 2 | 1 | | | | | | | 6 |
| Inflammation of " | | 2 | | | | | | 1 | | 1 | | | | | | | | 2 |
| Menorrhagia | | 1 | | | | | | | | | | 1 | | | | | | 1 |
| Onanism | 1 | | | | | | | 1 | | | | | | | | | | 1 |
| Phlegmasia dolens | | 1 | | | | | | | | 1 | | | | | | | | 1 |
| Puerperal Fever | | 6 | | | | | | | 4 | 2 | | | | | | | | 6 |
| Ovarian Tumour | | 1 | | | | | | | | 1 | | | | | | | | 1 |
| | 1 | 23 | | | | | | 2 | 7 | 11 | 2 | 1 | 1 | | | | | 24 |

### 9. *Organs of Locomotion.*

| | Male. | Female. | Under 1 yr. | 1 to 2. | 2 to 5. | 5 to 10. | 10 to 15. | 15 to 20. | 20 to 30. | 30 to 40. | 40 to 50. | 50 to 60. | 60 to 70. | 70 to 80. | 80 to 90. | 90 to 100. | 100 to 110. | Total. |
|---|---|---|---|---|---|---|---|---|---|---|---|---|---|---|---|---|---|---|
| Disease of Spine | 2 | | 1 | 1 | | | | | | | | | | | | | | 2 |
| Rheumatism | 1 | 5 | | | | | | | | 3 | | 1 | | 2 | | | | 6 |
| Spinal Irritation | | 1 | 1 | | | | | | | | | | | | | | | 1 |
| Spina Bifida | 1 | | 1 | | | | | | | | | | | | | | | 1 |
| | 4 | 6 | 3 | 1 | | | | | | 3 | | 1 | | 2 | | | | 10 |

### 10. *Integumentary System.*

| | Male. | Female. | Under 1 yr. | 1 to 2. | 2 to 5. | 5 to 10. | 10 to 15. | 15 to 20. | 20 to 30. | 30 to 40. | 40 to 50. | 50 to 60. | 60 to 70. | 70 to 80. | 80 to 90. | 90 to 100. | 100 to 110. | Total. |
|---|---|---|---|---|---|---|---|---|---|---|---|---|---|---|---|---|---|---|
| Purpura Hemorrhagica | | 1 | 1 | | | | | | | | | | | | | | | 1 |
| Scorbutis | 3 | | | | | | | | | 1 | 1 | | 1 | | | | | 3 |
| | 3 | 1 | 1 | | | | | | | 1 | 1 | | 1 | | | | | 4 |

### 11. *Old Age.*

| | Male. | Female. | Under 1 yr. | 1 to 2. | 2 to 5. | 5 to 10. | 10 to 15. | 15 to 20. | 20 to 30. | 30 to 40. | 40 to 50. | 50 to 60. | 60 to 70. | 70 to 80. | 80 to 90. | 90 to 100. | 100 to 110. | Total. |
|---|---|---|---|---|---|---|---|---|---|---|---|---|---|---|---|---|---|---|
| Old Age | 17 | 45 | | | | | | | | | | | 8 | 13 | 29 | 10 | 2 | 62 |

12. *External Causes.*

| | Male. | Female. | Under 1 yr. | 1 to 2. | 2 to 5. | 5 to 10. | 10 to 15. | 15 to 20. | 20 to 30. | 30 to 40. | 40 to 50. | 50 to 60. | 60 to 70. | 70 to 80. | 80 to 90. | 90 to 100. | 100 to 110. | Total. |
|---|---|---|---|---|---|---|---|---|---|---|---|---|---|---|---|---|---|---|
| Burns | 7 | 2 | 1 | 1 | 1 | 1 | | | 2 | 2 | 1 | | | | | | | 9 |
| Casualties | 32 | 10 | 2 | | | 2 | 2 | 1 | 8 | 16 | 6 | 4 | | 1 | | | | 42 |
| Drowned | 53 | 2 | | | 2 | 13 | 9 | 7 | 5 | 5 | 8 | 3 | 3 | | | | | 55 |
| Fracture | 1 | | | | | | | | | | 1 | | | | | | | 1 |
| " of Arm | 2 | | | | | 1 | | | | 1 | | | | | | | | 2 |
| " Skull | 2 | | | | | | | 1 | | 1 | | | | | | | | 2 |
| " Leg | 2 | | | | | | | | 1 | | | 1 | | | | | | 2 |
| Intemperance | 20 | 12 | | | | | | | 4 | 14 | 8 | 3 | 1 | 2 | | | | 32 |
| Poison | 3 | 2 | 1 | | 1 | | | 2 | | | 1 | | | | | | | 5 |
| Suffocation | 5 | | 1 | | | | | | | | 4 | | | | | | | 5 |
| Suicide | 3 | | | | | | | | 1 | 1 | | 1 | | | | | | 3 |
| | 130 | 28 | 5 | 1 | 4 | 17 | 11 | 11 | 21 | 40 | 29 | 12 | 4 | 3 | | | | 158 |
| Still Born | 73 | 59 | 132 | | | | | | | | | | | | | | | 132 |
| Unknown | 60 | 64 | 44 | 9 | 11 | 4 | 2 | 2 | 12 | 12 | 16 | 8 | 2 | 2 | | | | 124 |

TABLE NO. 3.

*Deaths for the Third Quarter of 1854, at fifteen distinct periods of life.*

| | |
|---|---|
| Under 1 year, . . . . . . | 1185 |
| 1 to 2 . . . . . . . | 699 |
| 2 to 5 . . . . . . . | 337 |
| 5 to 10 . . . . . . . | 140 |
| 10 to 15 . . . . . . . | 79 |
| 15 to 20 . . . . . . . | 99 |
| 20 to 30 . . . . . . . | 450 |
| 30 to 40 . . . . . . . | 417 |
| 40 to 50 . . . . . . . | 340 |
| 50 to 60 . . . . . . . | 234 |
| 60 to 70 . . . . . . . | 190 |
| 70 to 80 . . . . . . . | 134 |
| 80 to 90 . . . . . . . | 69 |
| 90 to 100 . . . . . . . | 22 |
| 100 to 110 . . . . . . . | 4 |
| | 4399 |
| Still Born . . . . . . | 132 |
| Total, | 4531 |

Included within the above table, were 393 from the Blockley Alms House; 242 Blacks, and 25 from the country, as follows:

| | July. | August. | September. | Total. |
|---|---|---|---|---|
| Almshouse, . . | 180 | 138 | 75 | 393 |
| Blacks, . . . . | 90 | 92 | 60 | 242 |
| Country, . . | 13 | 10 | 2 | 25 |
| Total, | | | | 660 |

